# Venous Thromboembolism in Transgender and Gender Diverse Individuals on Estrogen-Based Gender-Affirming Hormone Therapy

**DOI:** 10.3390/jcm15114166

**Published:** 2026-05-28

**Authors:** Sofia Burgoon, Hayley Cunningham, Heather R. Batchelder, Quinnette Jones, Carly E. Kelley, Sargam Kapoor

**Affiliations:** 1 Department of Family Medicine & Community Health, Duke University, Durham, NC 27710, USAheather.batchelder@duke.edu (H.R.B.);; 2 Duke Center for Health Policy & Inequities Research, Duke University, Durham, NC 27710, USA; 3 Division of Endocrinology, Metabolism, and Nutrition, Department of Medicine, Duke University, Durham, NC 27710, USA; 4 Division of Hematology, Department of Medicine, Duke University, Durham, NC 27710, USA; sargam.kapoor@duke.edu

**Keywords:** venous thromboembolism, transgender, gender-affirming hormone therapy, estrogen, thrombosis

## Abstract

**Background:** The use of estrogen-based gender-affirming hormone therapy (E-GAHT) has been associated with an increased risk of venous thromboembolism (VTE), but much of the evidence originates from data on cisgender women and from cohorts of transgender and gender diverse (TGD) individuals treated with older estrogen or estrogen/progesterone preparations, often at higher doses. Data on VTE risks associated with more modern E-GAHT regimens in TGD populations are scarce. **Methods:** A retrospective cohort study of adult TGD individuals who received E-GAHT within the Duke University Health System between January 1996 and June 2025 was conducted. The Duke Enterprise Data Unified Content Explorer (DEDUCE), a Duke electronic medical record search tool, was utilized to identify a cohort of TGD individuals who were prescribed E-GAHT. From this cohort, individuals who experienced a VTE during E-GAHT exposure were identified. Demographic characteristics and comorbidities were compared between the overall study cohort and those who experienced VTE using the SlicerDicer tool within Epic, supplemented by manual chart review. **Results:** Among 1173 adult TGD individuals prescribed E-GAHT, 16 (1.4%) experienced a VTE. Of these, 11 (68.8%) experienced a pulmonary embolism (PE with/without deep vein thrombosis [DVT]) and five (31.3%) experienced a DVT alone. Among the 16 patients with VTE, six (37.5%) had a transient surgical risk factor prior to VTE, three (18%) had significant non-surgical risk factors, and one (6%) had cancer. The remaining six (37.5%) patients experienced an unprovoked VTE. Patients with VTE were significantly older than the general population of TGD adults and were significantly more likely to experience hypertension, hyperlipidemia, and type 2 diabetes mellitus, compared to TGD patients without VTE. **Conclusions:** In this retrospective cohort, the proportion of TGD individuals on E-GAHT with VTE was lower than previously reported in the literature. Most events occurred in the presence of other established risk factors, suggesting that E-GAHT itself may confer a lower VTE risk than previously assumed. Larger prospective studies that evaluate both estrogen-specific and patient-specific risk factors are needed to clarify VTE risk in this population.

## 1. Introduction

Estrogen-based gender affirming hormone therapy (E-GAHT) is an essential treatment modality for the mental and physical health of transgender and gender diverse (TGD) individuals [1,2]. In 2024, 1.3% of adults in the United States identified as TGD, representing a notable increase from 0.6% in 2021 [3]. Access to gender affirming healthcare has been linked to improved quality of life and decreased risk of self-harm [4,5,6]. However, as with any medical therapy, E-GAHT use involves potential risks, including venous thromboembolism (VTE) [7]. VTE poses a significant public health burden with varying rates worldwide and up to 900,000 VTE events in the United States each year [8,9,10]. In addition to geographic variation, likely due to underlying genetic predispositions, a variety of patient-related and iatrogenic risk factors can modify VTE risk in variable ways [11]. An understanding of the incremental risk of VTE with E-GAHT, if any, is therefore critical to inform and support gender affirming care, a cornerstone of the management of TGD individuals.

Early observational data suggested an elevated risk of VTE among transgender women receiving E-GAHT, particularly in the context of older oral estrogen formulations. A 1997 Dutch cohort study of transgender women receiving E-GAHT found an approximately 20-fold increased incidence of VTE [12]. A subsequent Dutch cohort study of 2517 transgender women receiving E-GAHT between 1972 and 2015 found a higher incidence of VTE compared to cisgender women (standardized incidence ratio [SIR] 5.52) and cisgender men (SIR 4.55) in age-adjusted comparisons [7]. In a U.S.-based electronic medical record cohort from Kaiser Permanente in Georgia and California (index dates 2006–2014, followed through 2016), transgender women on E-GAHT experienced an increased VTE incidence, compared with cisgender men (hazard ratio [HR] 1.9) and cisgender women (HR 2.0) [13]. In this cohort, VTE risk became more pronounced with longer duration of E-GAHT. A 2021 systematic review of 22 retrospective observational studies reported that the pooled VTE incidence in transgender women on E-GAHT was 2.7% compared to 1.7% in cisgender women receiving hormone replacement therapy (HRT) [14]. Notably, most TGD participants in the included studies received oral estrogen formulations, such as conjugated estrogens, estradiol valerate, or ethinyl estradiol, reflecting historical prescribing practices, when estrogen type and route of administration differed from contemporary gender-affirming regimens [14].

More recent analyses suggest that VTE prevalence in TGD populations receiving E-GAHT may be lower than earlier reports implied. A 2021 meta-analysis including over 11,000 transgender women found an overall VTE prevalence of approximately 2%, with higher risk observed in older individuals and those with longer duration of therapy, groups more likely to have used ethinyl estradiol [15]. Similarly, in a cohort of 676 TGD individuals receiving oral 17-β-estradiol between 2008 and 2016, only one individual (0.15%) experienced VTE [16]. This apparent shift coincides with updated GAHT guidelines favoring modern estradiol-based regimens [17]. Contemporary E-GAHT regimens rely on 17-β-estradiol and estradiol valerate, bioidentical estrogens that more closely mimic endogenous hormones, generally prescribed at lower doses than historical preparations, and considered less thrombogenic [15]. Current practice also emphasizes titrating therapy to achieve physiologic estradiol concentrations of approximately 100–200 pg/mL rather than relying on fixed-dose regimens modeled after oral contraceptives or menopausal hormone therapy [18,19,20].

Our study sought to assess the number of VTE events in a large, single-institution cohort of TGD individuals on E-GAHT and to identify additional factors that may influence thrombotic risk in this population.

## 2. Materials and Methods

### 2.1. Study Design and Setting

A retrospective cohort study was conducted using the Data Driven Electronic Unified Cohort Explorer (DEDUCE) platform (version 8.4 (2314)) to identify TGD individuals exposed to E-GAHT within the Duke University Health System (DUHS), Durham, NC, USA.

### 2.2. Study Population

**Figure 1:**
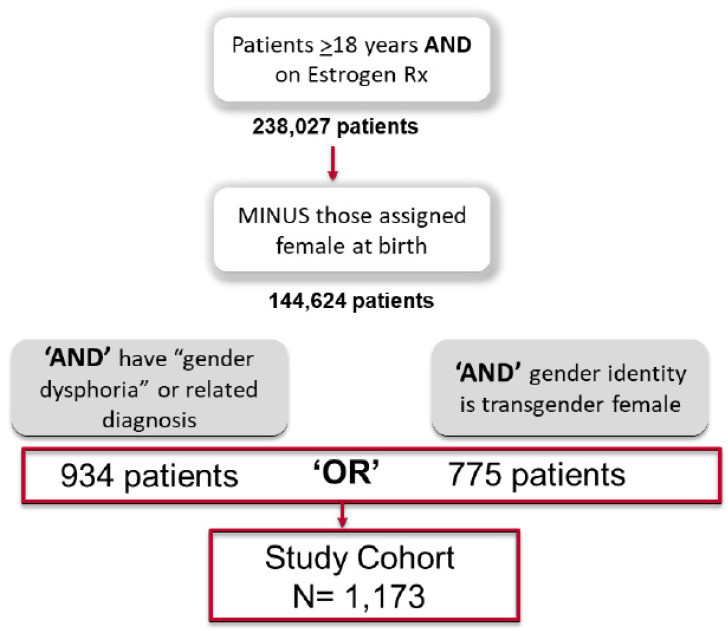
Cohort identification of transgender and gender diverse individuals prescribed estrogen-based gender affirming hormone therapy.

Patients were included in the study cohort if they met the following criteria: (1) were prescribed E-GAHT within DUHS between January 1996 and June 2025; (2) were 18 years or older when prescribed E-GAHT; and (3) were not assigned female sex at birth (Figure 1).

### 2.3. Data Collection and Definitions

TGD individuals were identified using a combination of demographic data (patient- or provider-reported gender identity and sex assigned at birth) and ICD 9/10 codes (transsexualism, 64.0; gender dysphoria, F64.1; other gender identity disorders, F64.8). E-GAHT exposure was identified through structured medication prescribing data from the electronic medical record.

TGD patients with VTE were identified using ICD 9/10 diagnostic codes for deep venous thrombosis (DVT; ICD10 I82, ICD9 415) and/or pulmonary embolism (PE; ICD10 I26, ICD9 415). Charts of identified individuals were then independently reviewed via keyword searches of clinical notes to confirm TGD status and a diagnosis of VTE while receiving E-GAHT. The most recent E-GAHT prescription prior to the VTE event was recorded.

The following demographic variables were collected: the age at time of VTE (or age at the time of data collection for those without documented VTE), patient-reported race and ethnicity, body mass index (BMI) at the time closest to cohort entry, smoking history, Human Immunodeficiency Virus (HIV) status, and cardiovascular comorbidities (hypertension, hyperlipidemia, and diabetes mellitus). Data on provoking risk factors for VTE were collected, and the VTE event was classified as either provoked (when preceded by risk factors for VTE) or unprovoked in accordance with the International Society on Thrombosis and Hemostasis guidance on categorization of VTE events [21]. Major transient risk factors included surgery, trauma, hospitalization, or immobilization. Minor transient risk factors included reduced mobility, medical illness or infection, or persistent risk factors such as cancer. E-GAHT route and dose at the time of VTE were identified, and the duration from E-GAHT initiation to the first VTE event was calculated in years. Presence of inherited and/or acquired thrombophilia was recorded if present in the medical record.

### 2.4. Statistical Analysis

Descriptive statistics were performed to obtain median age, ethnicity, race, mean BMI, smoking status, and diagnoses of hypertension, hyperlipidemia, diabetes mellitus type 2 (DM2), and HIV between the VTE and non-VTE groups. One-sample t-tests were used to compare the mean age and BMI of the VTE group with those of the total adult TGD population. Exploratory odds ratios and Fisher’s exact tests were calculated and used to compare ethnicity, race, smoking status (current), and comorbidities (hypertension, hyperlipidemia, DM 2, and HIV) between the VTE and non-VTE groups. As complete data were not available for the non-VTE sample, summary statistics were used to calculate the exploratory odds ratio and Fisher’s exact tests. Regression modeling was not performed due to the low event count in the VTE group. Analyses were conducted using Stata 18.5 software.

### 2.5. Ethics Statement

This study was granted a permanent ethical exemption by the Duke University Institutional Review Board (Protocol #00116264) due to its retrospective design.

## 3. Results

A total of 1173 adult TGD individuals were prescribed E-GAHT within DUHS between January 1996 and June 2025. Of these, 16 patients (1.4%) developed VTE while on E-GAHT.

### 3.1. Characteristics of the VTE Subgroup

Among the 16 patients with VTE, 10 were provoked (62.5%): six (37.5%) had a transient surgical risk factor prior to VTE, three (18%) had non-surgical risk factors (two with prolonged hospitalization and one with bacteremia), and one (6%) had cancer. The remaining six (37.5%) patients experienced an unprovoked VTE.

Nine patients (56%) with VTE were prescribed oral estrogen, three (18%) transdermal, three (18%) intramuscular, and one (6%) received estrogen by an unknown route (Table 1). Among the six individuals with unprovoked VTE, two were prescribed oral 17-β-estradiol, one was prescribed oral conjugated equine estrogen, two were prescribed intramuscular estrogen, and for one individual, the route was unknown. None were prescribed transdermal estrogen. Serum estradiol levels at the time of VTE diagnosis were not available in the electronic medical record for any of these patients. None of the individuals in the VTE subgroup had a known thrombophilia.

Mean duration from E-GAHT start to the first VTE event was 7.8 years (range 0.2 to 19 years) among all individuals with a VTE, and 7.9 years (range 1.5 to 12.5 years) for those with an unprovoked VTE.

### 3.2. Comparison Between the Total TGD Population, the Non-VTE Subgroup, and the VTE Subgroup

The total TGD population (N = 1173) had a median age of 29 years, 101 (8.61%) reported Hispanic ethnicity, 881 (75.11%) reported race as White, and 153 (12.96%) reported race as Black; mean BMI was 28.2 kg/m^2^, and 99 (8.43%) were current smokers (Table 2). The proportions of individuals in the total TGD population with comorbidities were 9.12% (N = 107) for hypertension, 10.66% (N = 125) for hyperlipidemia, 4.69% (N = 55) for DM2, and 5.37% (N = 63) for HIV.

For the non-VTE subgroup (N = 1157), 100 (8.64%) reported Hispanic ethnicity, 869 (75.11%) reported race as White, and 150 (12.96%) reported race as Black, with 96 (8.30%) reporting that they were current smokers (Table 2). The proportions of individuals in the non-VTE cohort with comorbidities were 8.56% (N = 99) for hypertension, 10.20% (N = 118) for hyperlipidemia, 4.41% (N = 51) for DM2, and 5.27% (N = 61) for HIV.

The VTE subgroup (N = 16) had a median age of 50 years, one (6.25%) reported Hispanic ethnicity, 75% (N = 12) reported race as White, and 18.75% (N = 3) as Black. The mean BMI was 31.19 kg/m^2^, and three (18.75%) were current smokers. Proportions of individuals with chronic comorbidities in the VTE subgroup were 50.0% (N = 8) for hypertension, 43.75% (N = 7) for hyperlipidemia, 25.0% (N = 4) for DM2, and 12.5% (N = 2) for HIV. While none of the 16 VTE patients had a documented thrombophilia diagnosis, four non-VTE individuals (0.3% of the overall cohort) had a known thrombophilia (Factor V Leiden).

The mean age of the VTE subgroup [50.63 years, Standard Deviation (SD) = 14.23] was significantly higher than the total TGD population mean (t(15) = 4.96, *p* < 0.001); however, the mean BMI for the VTE subgroup (31.19, SD = 6.81) was not significantly higher than the total TGD population mean (t(15) = 1.75, *p* = 0.101) (Table 2). The VTE sub-group was significantly more likely to experience hypertension (OR: 10.69, 95% CI: 3.40–33.30, *p* < 0.001), hyperlipidemia (OR = 6.85, 95% CI: 2.12–21.03, *p* = 0.001), and DM2 (OR: 7.23, 95% CI: 1.64–24.85, *p* = 0.005) compared to the non-VTE group. However, the VTE sub-group was not significantly more likely to be HIV positive (OR: 2.57, 95% CI: 0.28–11.56, *p* = 0.211), identify as Hispanic/Latino (OR: 0.70, 95% CI: 0.02–4.68, *p* = 1.000), identify as a race other than White (OR: 1.01, 95% CI: 0.23–3.35, *p* = 1.000), or be a current smoker (OR: 2.55, 95% CI: 0.46–9.50, *p* = 0.146) compared to the non-VTE group (Table 2).

## 4. Discussion

This retrospective cohort study from a large academic health care system found a smaller cumulative proportion of VTE (1.4%) among TGD individuals receiving E-GAHT. This observed proportion is lower than that reported in earlier studies, which have described VTE rates as high as 6% and substantially elevated relative risk compared with cisgender male populations [12,18,22,23,24]. However, in the absence of a comparator group and standardized follow-up time, this finding should be interpreted with caution and not as a direct estimate of relative risk.

VTE risk in the general population is influenced by multiple established patient-related and treatment-related factors, including age, body mass index, smoking, immobility, and comorbidities such as atherosclerosis, malignancy, inflammatory bowel disease, and congestive heart failure [11,25,26]. In our cohort, individuals who developed VTE were older and more likely to have cardiometabolic comorbidities, including hypertension, hyperlipidemia, and DM2, and frequently had identifiable provoking factors such as recent surgery or malignancy. These findings suggest that the occurrence of VTE in this population may be largely driven by established risk factors rather than E-GAHT exposure alone. Despite the low number of events in the VTE group and an exploratory analysis yielding odds ratios with wide confidence intervals, our results are hypothesis-generating and consistent with prior literature, which demonstrated increased odds of VTE among TGD individuals with metabolic comorbidities and did not identify an independent association between estrogen use and VTE after adjustment [27].

However, given the retrospective design and absence of a non-E-GAHT comparator group, it is not possible to determine whether observed VTE events are attributable to hormone therapy or to underlying comorbidities. It is well established that cardiovascular comorbidities play an important role as risk factors for incident and recurrent VTE, and evidence suggests that management of cardiovascular and metabolic risk factors is associated with improved VTE outcomes [11,28,29].

None of the VTE patients in our cohort had a known inherited or acquired thrombophilia; out of the entire cohort, four patients had Factor V Leiden mutations. Thrombophilia has been shown to have a synergistic effect on VTE risk with combined oral contraceptive use in cisgender women [30]. The effect of underlying thrombophilia on VTE risk with E-GAHT in the TGD population remains, however, poorly understood and warrants further study [31,32].

Formulation of estrogen appears to influence VTE risk in TGD individuals [8,32]. Importantly, none of the unprovoked VTE events in our cohort occurred while receiving transdermal estrogen. This observation is consistent with literature from cisgender populations suggesting that transdermal estrogen is less thrombogenic than oral formulations and supports the safety of transdermal E-GAHT routes [33]. While caution is warranted in extrapolating these results, transdermal administration is known to bypass first-pass hepatic metabolism, potentially resulting in a lower impact on coagulation factors and, consequently, a reduced thrombotic risk [33,34].

The mean latency to the first VTE event in our cohort was approximately 7.8 years, highlighting that VTE risk in this population can persist beyond the early E-GAHT phases, and possibly suggesting a role of advancing age and a corresponding increase in comorbidity burden over time. This is consistent with prior literature, which has shown increased VTE risk with longer duration of E-GAHT [13]. In contrast, VTE risk in cisgender women using combined oral contraceptives is highest during the first 6–12 months of use and subsequently plateaus but remains elevated with continued therapy [17]. This prolonged latency to the first VTE noted by our group, therefore, underscores the need for long-term vigilance in TGD individuals on E-GAHT and warrants further research to evaluate the longitudinal risk of VTE in TGD individuals on E-GAHT [13,14].

This study has several limitations. Interpretation of these findings is limited by the retrospective, single-center design, absence of a non–E-GAHT comparator group, and inconsistent follow-up time across individuals, which did not permit estimation of relative risk or incidence. Additionally, reliance on retrospectively collected medical record data led to incomplete and inconsistent documentation, including missing serum estradiol levels for the VTE cohort. While there is no known established association between estradiol levels and VTE risk, lack of these data limits the study’s ability to track medication compliance and the dose–response relationship with VTE risk [35]. Smoking history, immobilization, and adherence to E-GAHT were also incompletely captured in the medical record, representing a key limitation given their direct relevance to VTE risk estimation [36,37].

Another limitation is the limited sensitivity of using ICD codes and keyword searches for identifying outcomes, which may result in misclassification of VTE events. Moreover, it is important to note that diagnostic imaging, including CT pulmonary angiography and venous duplex ultrasound, was performed only in the context of clinical suspicion for VTE. As a result, we cannot exclude the possibility that some cases of asymptomatic or subclinical VTE may have gone undetected, leading to an underestimation of the true burden of VTE in our cohort.

Finally, in this single-institution study, our patient population was primarily White and received care from a large academic medical center in the southeastern US. This limits the generalizability of the study results to other care settings, including communities with potentially more diverse populations.

Despite these limitations, this study provides important data on VTE risk among TGD individuals on modern E-GAHT and highlights the importance of comprehensive risk profiling and optimization of modifiable risk factors. Clinicians prescribing E-GAHT should consider not only the hormonal regimen but also traditional patient and treatment-specific risk factors (age, BMI, smoking history, metabolic disease, cancer, surgery, and mobility) when prescribing E-GAHT. The potential impact of estrogen formulation and route (transdermal, oral, or intramuscular) on VTE risk, as well as the role of circulating estradiol levels and underlying thrombophilia, warrants further investigation.

## 5. Conclusions

In this retrospective study, the proportion of VTE among TGD individuals on E-GAHT was 1.4%. Most events occurred in the presence of at least one other established risk factor. Additionally, larger prospective studies that evaluate both estrogen-specific and patient-specific risk factors are needed to better understand VTE risk in this population.

## Figures and Tables

**Table 1:** Estrogen regimen in patients with unprovoked venous thromboembolism.

Patient	Estrogen Preparation and Dosing	Age at VTE (years)	Age at GAHT Initiation (Years)	BMI (kg/m^2^)
1	PO Estradiol 4 mg daily	54	47	37.0
2	PO Conjugated Estrogen 1.25 mg BID	47	34	28.13
3	PO Estradiol 2 mg daily	32	29	38.23
4	IM Estradiol valerate 20 mg weekly	53	42	29.3
5	IM Estradiol cypionate 3 mg weekly	49	48	32.24
6	Unknown	50	40	21.67

VTE: venous thromboembolism; PO: oral; IM: intramuscular; GAHT: gender-affirming hormone therapy; BMI: body mass index.

**Table 2:** Baseline characteristics of transgender and gender diverse individuals on estrogen-based gender affirming hormone therapy and in the subgroup with venous thromboembolism.

Characteristics	Total TGD Population (N = 1173) N (%)	Non-VTE Cohort (N = 1157) N (%)	VTE Cohort (N = 16) N (%)	t-Value (*p*-Value)	OR	95% CI	*p*-Value
Median age (range) in years	29 (18–87)	-	50 (30–80)	4.96 (<0.001)	-	-	-
Mean BMI (kg/m^2^)	28.2	-	31.19 (6.81)	1.75 (0.101)	-	-	-
Ethnicity							
Hispanic	101 (8.61%)	100 (8.64%)	1 (6.25%)	-	0.70	0.02–4.68	1.000
Not Hispanic/Latino	964 (82.18%)	950 (82.11%)	14 (87.5%)	-			
Unknown	108 (9.21%)	107 (9.25%)	1 (6.25%)	-			
Race *							
White	881 (75.11%)	869 (75.11%)	12 (75%)	-	1.01	0.23–3.35	1.000
Black	153 (13.04%)	150 (12.96%)	3 (18.75%)	-			
Native American	15 (1.28%)	14 (1.21%)	1 (6.25%)	-			
Asian	30 (2.56%)	30 (2.59%)	0 (0%)	-			
Other	41 (3.50%)	41 (3.54%)	0 (0%)	-			
Not reported	71 (6.05%)	71 (6.14%)	0 (0%)	-			
Two or more	15 (1.28%)	15 (1.30%)	0 (0%)	-			
Smoking at the time of data collection							
Never	817 (69.65%)	806 (69.66%)	11 (68.75%)	-	2.55	0.46–9.50	0.146
Former	221 (18.84%)	219 (18.93%)	2 (12.50%)	-			
Current	99 (8.43%)	96 (8.30%)	3 (18.75%)	-			
Unknown	36 (3.07%)	36 (3.11%)	0 (0%)	-			
Hypertension	107 (9.12%)	99 (8.56%)	8 (50.0%)	-	10.69	3.40–33.30	<0.001
Hyperlipidemia	125 (10.66%)	118 (10.20%)	7 (43.75%)	-	6.85	2.12–21.03	0.001
DM2	55 (4.69%)	51 (4.41%)	4 (25.0%)	-	7.23	1.64–24.85	0.005
HIV positive	63 (5.37%)	61 (5.27%)	2 (12.5%)	-	2.57	0.28–11.56	0.211

BMI: body mass index; DM2: diabetes mellitus type 2; HIV: human immunodeficiency virus OR: Odds Ratio; VTE: venous thromboembolism. * >1 race category able to be selected by patients.

## Data Availability

The original contributions presented in this study are included in the article. Further inquiries can be directed to the corresponding author.
